# Optimal cardiac strategy based on the history of myocardial infarction in type 2 diabetic patients with coronary artery disease

**DOI:** 10.1038/s41598-019-39857-0

**Published:** 2019-03-05

**Authors:** Tetsuro Tsujimoto, Hiroshi Kajio

**Affiliations:** Department of Diabetes, Endocrinology, and Metabolism, National Center for Global Health and Medicine, Tokyo, Japan

## Abstract

The aim of this study was to evaluate the association between the cardiac treatment strategy and cardiac event risk in type 2 diabetic patients with coronary artery disease (CAD) based on the history of myocardial infarction. Using Bypass Angioplasty Revascularization Investigation 2 Diabetes (BARI 2D) trial data, a Cox proportional hazard model was used for calculating hazard ratios (HRs) for major cardiac events in patients receiving early revascularization or intensive medical therapy. Patients without (*n* = 1,557) and with myocardial infarction (*n* = 736) were separately analyzed. In patients without myocardial infarction, risk of major cardiac events was similar for percutaneous coronary intervention and intensive medical therapy groups, whereas it was significantly lower in the coronary artery bypass grafting group than in the intensive medical therapy group (HR: 0.48, 95% confidence interval [95%CI]: 0.30–0.76, *P* = 0.002). Conversely, in patients with myocardial infarction, risk of major cardiac events was significantly higher in the early revascularization group than in the intensive medical therapy group (HR: 1.47, 95%CI: 1.03–2.11, *P* = 0.03). In type 2 diabetic patients with CAD, benefits of early revascularization were observed only in those without previous myocardial infarction. For patients with previous myocardial infarction, intensive medical therapy exerted superior benefits.

## Introduction

The number of type 2 diabetic patients is increasing worldwide^1,2^; an appropriate management of this disease is vital for preventing the complications of diabetes. A good glycemic control decreases the risk of microvascular disease^3^. Conversely, recent trials have reported that intensive glycemic control may not prevent cardiovascular events, such as coronary artery disease (CAD) and stroke^4–6^. Although some diabetic patients suffer from CAD despite good glycemic control, the optimal treatment strategy for these patients remains unclear^7^. The evidence regarding treatment strategies for diabetic patients with CAD was gathered from the Bypass Angioplasty Revascularization Investigation 2 Diabetes (BARI 2D) trial, which revealed that risks of mortality and a composite endpoint comprising nonfatal myocardial infarction, nonfatal stroke, or all-cause death were not significantly different between patients receiving revascularization combined with medical therapy and those receiving medical therapy alone. However, it remains unknown whether similar results would be obtained for patients with different conditions. Particularly, early revascularization may be harmful for diabetic patients with CAD who already have a history of myocardial infarction despite being beneficial for those without previous myocardial infarction. Thus, the aim of this study was to evaluate the association between the cardiac treatment strategy and cardiac event risk in type 2 diabetic patients with CAD based on the history of myocardial infarction.

## Methods

### Study design and patients

We used data from the BARI 2D trial, which included 2,368 type 2 diabetic patients with CAD^7–11^. Briefly, the BARI 2D trial was a randomized clinical trial using a 2 × 2 factorial design: (1) early revascularization combined with intensive medical therapy versus intensive medical therapy alone and (2) providing more endogenous or exogenous insulin versus reducing insulin resistance. Randomization was stratified according to the revascularization method (percutaneous coronary intervention [PCI] or coronary artery bypass grafting [CABG])^7–11^. The diagnosis of CAD was based on angiography records: classic angina and a major epicardial coronary artery stenosis ≥70% or a major epicardial coronary artery stenosis ≥50% associated with a positive stress test. Patients were excluded if they had required immediate coronary revascularization, undergone revascularization within 1 year prior to this study, had glycated hemoglobin >13.0%, hepatic disease, serum creatinine >2 mg/dL, congestive heart failure (class III or IV), or left main coronary artery stenosis ≥50%. Because patients aged 80 years or older (*n* = 38) were regarded as patients aged 80 years, they were excluded from the present analyses. Additionally, patients without information on history of myocardial infarction (*n* = 37) were excluded, leading to a sample of 2,293 subjects. This study was approved by the National Center for Global Health and Medicine review board, and the National Heart, Lung, and Blood Institute approved our use of the trial data. This study was conducted in accordance with the Declaration of Helsinki. All methods were conducted in accordance with the relevant guidelines and regulations. Because this study was a post-hoc analysis of the BARI 2D trial, this study received the ethical approval for the use of an opt-out method of obtaining informed consent. The patient data were anonymized by the National Heart, Lung, and Blood Institute before our use of data.

### Outcome evaluation

The primary outcome was the occurrence of major cardiac events, which was a composite endpoint comprising nonfatal myocardial infarction and cardiac death. The secondary outcomes were the occurrence of fatal or nonfatal myocardial infarction—including spontaneous, silent, and procedure-related events^7,12^—cardiac death, and all-cause death. Spontaneous myocardial infarction was diagnosed on the basis of the doubling of cardiac biomarkers such as creatine kinase MB or troponin and evidence of ischemia evaluated from symptoms, electrocardiography, or imaging^7,12^. Silent myocardial infarction was diagnosed on the basis of a two-grade Q-wave change on routine electrocardiography, according to the Minnesota code^7,12^. Cardiac death included death from myocardial infarction, congestive heart failure, cardiogenic shock, and sudden cardiac death occurring instantaneously or within 60 min after the onset of cardiac symptoms. It also included death within 30 days or within same hospitalization for a cardiac procedure such as PCI, CABG, and diagnostic angiogram. The evaluation of outcomes of the BARI 2D trial was previously reported in detail^7,12^, and the adjudication and classification of the endpoint data were performed by an independent Mortality and Morbidity Classification Committee. Patients were evaluated monthly during the first 6 months and every 3 months thereafter, up to a maximum of 5 years.

### Statistical analysis

Categorical and continuous variables were compared using the chi-squared test and the *t*-test, respectively. The continuous variables were tested using a histogram, which showed a normal distribution. The event rates of outcomes were calculated in patients with or without history of myocardial infarction, and Kaplan–Meier survival curves were generated^13–15^. Hazard ratios (HRs) and 95% confidence intervals (CIs) for outcomes were calculated by Cox proportional hazard models^13,16^. The comparison between the early revascularization and medical therapy groups was performed separately in patients with and without history of myocardial infarction. Further analyses assessed the HRs for primary or secondary outcomes in the CABG and PCI groups and compared them with those in the intensive medical therapy group. We tested the proportional hazards assumption using graphical and scaled Schoenfeld residual methods^17^. The proportional hazards assumption was met for the analyses in patients with history of myocardial infarction. Considering the assumptions might be violated in part of the analyses in patients without history of myocardial infarction, we performed an additional analysis considering cardiac treatment strategy as a time-varying variable in an extended Cox model^18^. To assess the effect modification of previous myocardial infarction on the association between cardiac treatment strategy and major cardiac events, we tested for the interaction between history of myocardial infarction and cardiac treatment strategy. The analysis included cardiac treatment strategy, history of myocardial infarction, and their interaction term in the Cox proportional hazard model. Major cardiac event risk following cardiac procedures was assessed by comparing the incidence of those events within 1 year after the follow-up in the revascularization, PCI, or CABG groups with the intensive medical therapy group in each patient with and without history of myocardial infarction.

Furthermore, the primary outcome was compared between subgroups, such as age (<65 or ≥65 years), gender (male or female), race (non-white or white), obesity (non-obesity or obesity), duration of diabetes (<10 or ≥10 years), glycated hemoglobin (<7% or ≥7%), and glycemic treatment. The body mass index was calculated by dividing the body weight (kg) by the square of height (m^2^). The definition of obesity was a body mass index ≥30 kg/m^2^. Interactions between the cardiac treatment strategy and these subgroups were investigated.

A level of *P* < 0.05 was considered statistically significant. We used Stata software (version 14.1; Stata Corp., College Station, Texas, USA) to analyze data.

## Results

### Baseline characteristics

The baseline characteristics of patients without (*n* = 1,557) or with history of myocardial infarction (*n* = 736) are presented in Table 1. In patients without and with history of myocardial infarction, the mean (±standard deviation) ages were 62.0 (±8.4) and 60.9 (±8.8) years, and the proportion of females were 31.5% and 25.5%, respectively. In patients without and with history of myocardial infarction, baseline characteristics were not significantly different between the early revascularization and intensive medical therapy groups.

**Table 1:** Baseline characteristics of patients with and without history of myocardial infarction*.

	Myocardial infarction (−)			Myocardial infarction (+)		
	Medical therapy	Revascularization	P value	Medical therapy	Revascularization	P value
	N = 778	N = 779		N = 372	N = 364	
Age (years)	62.2 (8.4)	61.8 (8.5)	0.41	60.8 (9.0)	61.0 (8.5)	0.69
Female sex (%)	31.6	31.3	0.89	25.0	26.1	0.73
Race and ethnicity (White, %)	70.1	69.8	0.92	71.5	70.6	0.78
Current smoker (%)	10.3	11.8	0.34	16.2	16.3	0.97
Education level (%)						
Less than high school	35.6	35.2	0.85	39.5	41.5	0.58
High school	24.2	22.8	0.51	18.8	19.2	0.89
More than high school	40.2	42.0	0.45	41.7	39.3	0.51
Physical activity (%)						
Sedentary	20.6	19.8	0.685	24.7	24.9	0.95
Mild	40.3	43.9	0.146	41.7	40.4	0.71
Moderate/strenuous	39.1	36.3	0.25	33.6	34.7	0.74
Body mass index (kg/m^2^)^†^	32.1 (5.7)	31.9 (6.0)	0.37	31.3 (5.8)	31.1 (5.4)	0.59
Diabetes duration ≥10 y (%)	43.4	43.8	0.88	42.4	40.0	0.50
Hypertension (%)	83.3	83.4	0.97	81.3	81.1	0.92
Hypercholesterolemia (%)	79.3	82.0	0.18	85.1	84.5	0.83
History of stroke/TIA (%)	9.0	7.6	0.30	11.8	13.5	0.50
History of chronic heart failure	5.3	5.5	0.83	7.7	10.3	0.20
Medications						
ACE-I (%)	64.1	62.3	0.47	68.7	67.2	0.66
ARB (%)	16.5	14.1	0.19	13.2	11.3	0.43
Calcium channel blocker (%)	33.7	31.1	0.27	31.8	27.3	0.17
Beta blockers (%)	67.7	69.8	0.36	83.3	79.9	0.23
Diuretics (%)	38.4	39.0	0.80	35.9	37.7	0.59
Statin (%)	72.3	71.6	0.74	81.4	81.0	0.90
Aspirin (%)	88.1	85.6	0.13	90.5	90.3	0.92
Biguanides (%)	55.2	56.2	0.68	54.0	48.6	0.14
Insulin (%)	30.0	27.4	0.26	26.6	28.0	0.66
Glycated hemoglobin (%)	7.7 (1.6)	7.7 (1.7)	0.97	7.7 (1.7)	7.6 (1.6)	0.43
Low-density lipoprotein (mg/dL)	98.9 (36.2)	96.6 (31.8)	0.20	93.7 (33.3)	92.6 (31.1)	0.67
High-density lipoprotein (mg/dL)	38.9 (10.1)	38.4 (10.3)	0.41	37.3 (10.1)	36.6 (10.0)	0.37
Estimated GFR (ml/min/1.73 m^2^)	71.8 (21.5)	72.7 (34.3)	0.51	73.3 (22.2)	72.3 (21.9)	0.53
Glycemic treatment assignment						
Insulin providing (%)	51.2	49.8	0.59	46.5	51.9	0.14

*Data are presented as number of participants, percent, or mean (standard deviation). Categorical and continuous variables were compared using the chi-squared test and *t*-test, respectively. P values were calculated by comparing each variable at baseline between the medical therapy and revascularization groups.

^†^Body mass index was calculated as weight in kilograms divided by the square of height in meters.

MI, myocardial infarction; TIA, transient ischemic attack; ACE-I, angiotensin-converting enzyme inhibitors; ARB, angiotensin II receptor blockers; GFR, glomerular filtration rate.

### Cardiac treatment strategy and risk of major cardiac events

**Figure 1:**
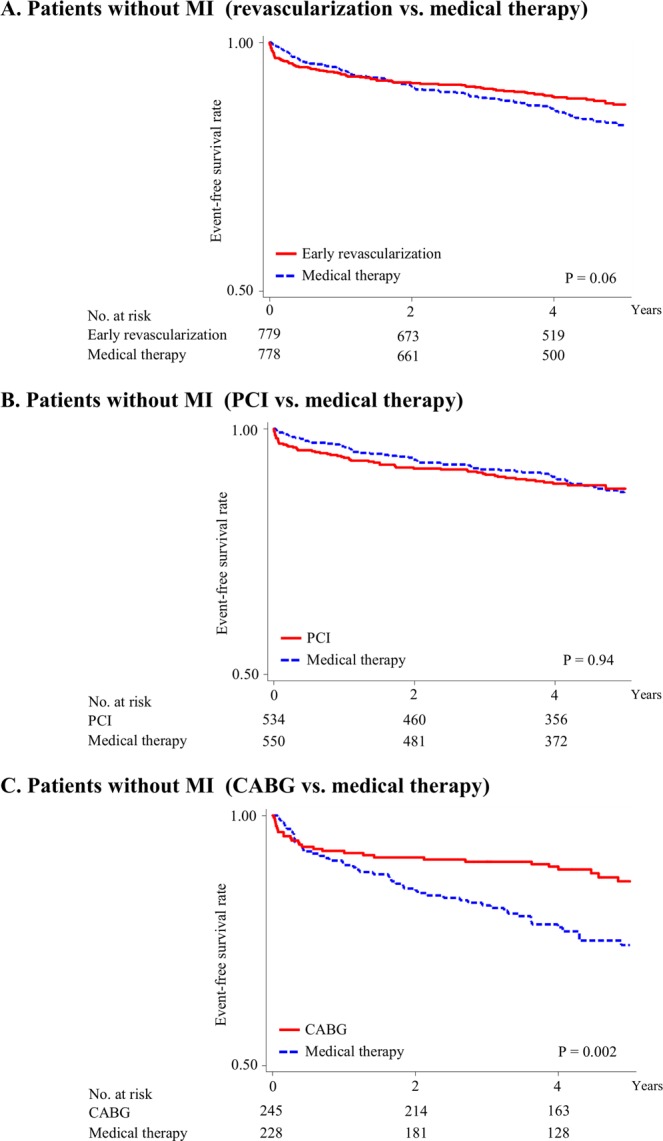
Kaplan–Meier survival curves for cardiac events in patients without history of myocardial infarction. Rates of freedom from major cardiac events: early revascularization vs. medical therapy (**A**), PCI vs. medical therapy (**B**), and CABG vs. medical therapy (**C**). Major cardiac events include cardiac death and nonfatal myocardial infarction. Cox proportional hazard analyses were performed to calculate hazard ratios and P values for major cardiac events in the revascularization group were compared to the medical therapy group in patients without history of myocardial infarction. PCI, percutaneous coronary intervention; CABG, coronary artery bypass graft surgery.

**Figure 2:**
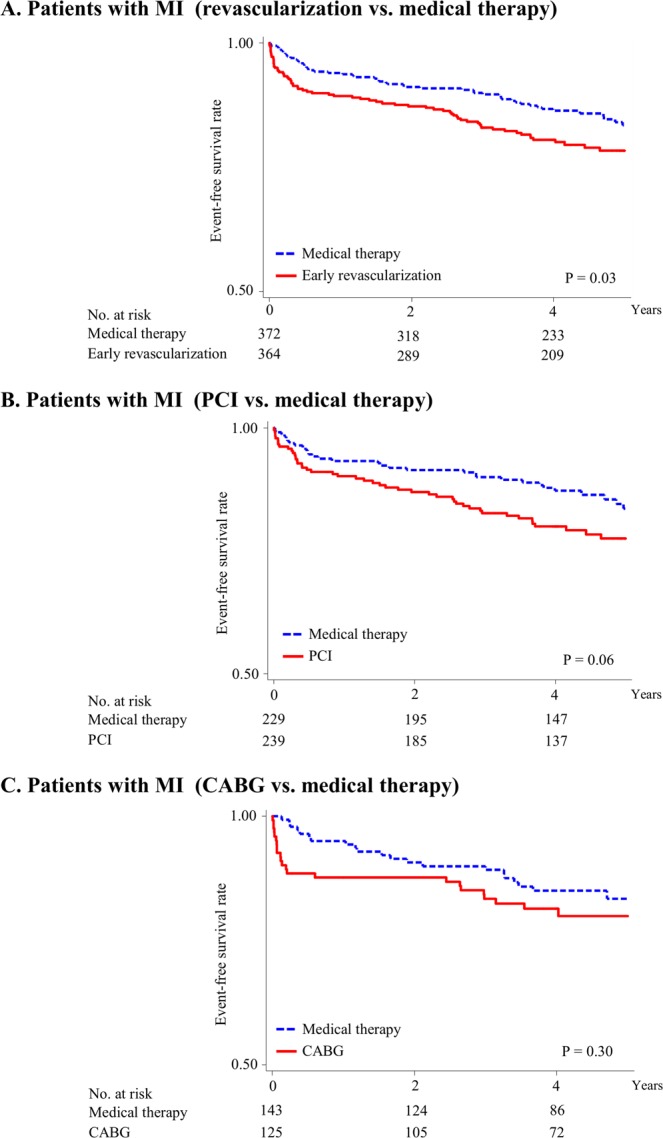
Kaplan–Meier survival curves for cardiac events in patients with history of myocardial infarction. Rates of freedom from major cardiac events: early revascularization vs. medical therapy (**A**), PCI vs. medical therapy (**B**), and CABG vs. medical therapy (**C**). Major cardiac events include cardiac death and nonfatal myocardial infarction. Cox proportional hazard analyses were performed to calculate hazard ratios and P values for major cardiac events in the revascularization group were compared to the medical therapy group in patients with history of myocardial infarction. PCI, percutaneous coronary intervention; CABG, coronary artery bypass graft surgery.

The overall mean (±standard deviation) follow-up period was 3.8 (±1.5) years: 3.9 (±1.5) and 3.6 (±1.6) years for patients without and with previous myocardial infarction, respectively. The Kaplan–Meier survival curves for major cardiac events in patients without and with previous myocardial infarction are shown in Figs 1 and 2, respectively. The event rates and HRs for major cardiac events, fatal or nonfatal myocardial infarction, cardiac death, and all-cause death in the early revascularization, PCI, CABG, and intensive medical therapy groups are shown in Table 2.

**Table 2:** Cardiac events and mortality in patients with and without history of myocardial infarction*.

	Myocardial infarction (−)			Myocardial infarction (+)		
	Medical therapy	Revascularization	P value	Medical therapy	Revascularization	P value
	N = 778	N = 779		N = 372	N = 364	
**Event**						
Major cardiac events						
No. of patients	112	87		52	70	
Event rate (per 1,000 person-year)	37.6	28.8		36.9	54.9	
Hazard Ratio (95% CI)	1.00 (ref)	0.77 (0.58–1.02)	0.06	1.00 (ref)	1.47 (1.03–2.11)	0.03
Myocardial infarction						
No. of patients	94	61		45	54	
Event rate (per 1,000 person-year)	31.6	20.2		32.0	42.4	
Hazard ratio (95% CI)	1.00 (ref)	0.64 (0.46–0.88)	0.007	1.00 (ref)	1.31 (0.88–1.95)	0.17
Cardiac death						
No. of patients	40	37		17	25	
Event rate (per 1,000 person-year)	11.4	10.7		10.4	16.0	
Hazard ratio (95% CI)	1.00 (ref)	0.94 (0.60–1.46)	0.77	1.00 (ref)	1.53 (0.83–2.84)	0.17
All-cause death						
No. of patients	112	87		52	70	
Event rate (per 1,000 person-year)	24.3	22.6		23.3	28.9	
Hazard ratio (95% CI)	1.00 (ref)	0.93 (0.68–1.27)	0.64	1.00 (ref)	1.24 (0.81–1.91)	0.32
	**Medical therapy**	**PCI**	**P value**	**Medical therapy**	**PCI**	**P value**
	**N** = **550**	**N** = **534**		**N** = **229**	**N** = **239**	
**Event**						
Major cardiac events						
No. of patients	61	59		31	47	
Event rate (per 1,000 person-year)	28.0	28.4		35.8	56.3	
Hazard Ratio (95% CI)	1.00 (ref)	1.01 (0.71–1.45)	0.94	1.00 (ref)	1.55 (0.98–2.43)	0.06
Myocardial infarction						
No. of patients	53	45		28	37	
Event rate (per 1,000 person-year)	24.3	21.7		32.3	44.3	
Hazard ratio (95% CI)	1.00 (ref)	0.89 (0.60–1.32)	0.55	1.00 (ref)	1.35 (0.83–2.20)	0.23
Cardiac death						
No. of patients	16	20		12	15	
Event rate (per 1,000 person-year)	6.4	8.3		12.0	14.6	
Hazard ratio (95% CI)	1.00 (ref)	1.31 (0.68–2.53)	0.41	1.00 (ref)	1.21 (0.57–2.58)	0.62
All-cause death						
No. of patients	61	59		31	47	
Event rate (per 1,000 person-year)	18.3	20.4		24.0	29.2	
Hazard ratio (95% CI)	1.00 (ref)	1.12 (0.75–1.68)	0.57	1.00 (ref)	1.22 (0.71–2.08)	0.47
	**Medical therapy**	**CABG**	**P value**	**Medical therapy**	**CABG**	**P value**
	**N** = **228**	**N** = **245**		**N** = **229**	**N** = **239**	
**Event**						
Major cardiac events						
No. of patients	51	28		21	23	
Event rate (per 1,000 person-year)	63.9	29.6		38.8	52.4	
Hazard Ratio (95% CI)	1.00 (ref)	0.48 (0.30–0.76)	0.002	1.00 (ref)	1.36 (0.75–2.46)	0.30
Myocardial infarction						
No. of patients	41	16		17	17	
Event rate (per 1,000 person-year)	51.4	16.9		31.4	38.7	
Hazard ratio (95% CI)	1.00 (ref)	0.34 (0.19–0.61)	<0.001	1.00 (ref)	1.25 (0.64–2.45)	0.51
Cardiac death						
No. of patients	24	17		5	10	
Event rate (per 1,000 person-year)	24.6	16.2		7.9	18.7	
Hazard ratio (95% CI)	1.00 (ref)	0.66 (0.35–1.23)	0.18	1.00 (ref)	1.29 (0.81–6.90)	0.11
All-cause death						
No. of patients	51	28		21	23	
Event rate (per 1,000 person-year)	39.9	27.6		22.1	28.1	
Hazard ratio (95% CI)	1.00 (ref)	0.69 (0.43–1.12)	0.13	1.00 (ref)	1.27 (0.62–2.64)	0.51

*Data are presented as number or hazard ratio (95% CI). Cox proportional hazard analyses were performed to calculate hazard ratios (95% confidence intervals), and P values for outcomes in the revascularization group were compared to the medical therapy group, separately in patients without and with history of myocardial infarction.

CI, confidence interval; PCI, percutaneous coronary intervention; CABG, coronary artery bypass graft surgery.

In patients without previous myocardial infarction, the risk of major cardiac events did not significantly differ between the early revascularization and intensive medical therapy groups (HR compared between the early revascularization and intensive medical therapy [ref] groups: 0.77, 95% CI: 0.58–1.02, *P* = 0.06, Fig. 1A). In addition, the risk of major cardiac events did not differ significantly between the PCI and intensive medical therapy groups (HR: 1.01, 95% CI: 0.71–1.45, *P* = 0.94, Fig. 1B), whereas it was significantly lower in the CABG group than in the intensive medical therapy group (HR: 0.48, 95% CI: 0.30–0.76, *P* = 0.002, Fig. 1C). The incidence of major cardiac events within 1 year of follow-up in patients without previous myocardial infarction was not significantly different between the early revascularization and intensive medical therapy groups (6.2% vs. 5.4%, respectively [*P* = 0.51]). The analysis using a time-varying model showed similar results.

Conversely, in patients with previous myocardial infarction, the risk of major cardiac events was significantly higher in the early revascularization group than in the intensive medical therapy group (HR: 1.47, 95% CI: 1.03–2.11, *P* = 0.03, Fig. 2A), and it was nonsignificantly higher in both the PCI and CABG groups than in the intensive medical therapy group (HR: 1.55, 95% CI: 0.98–2.43, *P* = 0.06 [Fig. 2B] and HR: 1.36, 95% CI: 0.75–2.46, *P* = 0.30 [Fig. 2C], respectively). A significant interaction was found between the cardiac treatment strategy and the previous history of myocardial infarction (*P* for interaction = 0.005). Similarly, the analysis limited to patients assigned *a priori* to the CABG stratum undergoing CABG as revascularization therapy showed a significant interaction between the cardiac treatment strategy (CABG or intensive medical therapy) and the previous myocardial infarction (*P* for interaction = 0.006). Incidence of major cardiac events within 1 year of follow-up in patients with previous myocardial infarction was significantly higher in the revascularization group than in the intensive medical therapy group (10.4% vs. 5.9%, respectively [*P* = 0.02]). In addition, in patients with previous myocardial infarction, incidence of major cardiac events was nonsignificantly higher in the PCI group than in the intensive medical therapy group (9.6% vs. 6.6%, respectively [*P* = 0.22]) and significantly higher in the CABG group than in the intensive medical therapy group (12.0% vs. 4.9%, respectively [*P* = 0.03]). The risk of major cardiac events after 1 year of follow-up in patients with previous myocardial infarction was not significantly higher in the revascularization, PCI, or CABG groups than in the intensive medical therapy group (HR: 1.19, 95% CI: 0.72–1.96, *P* = 0.48; HR: 1.59, 95% CI: 0.84–2.99, *P* = 0.15; and HR: 0.71, 95% CI: 0.30–1.68, *P* = 0.43, respectively).

The risk of fatal or nonfatal myocardial infarction in patients without previous myocardial infarction was significantly lower in the early revascularization group than in the intensive medical therapy group (HR: 0.64, 95% CI: 0.46–0.88, *P* = 0.007, eFig. 1A), where it was not significantly different between the PCI and intensive medical therapy groups (HR: 0.89, 95% CI: 0.60–1.32, *P* = 0.55, eFig. 1B), but was significantly lower in the CABG group than in the medical therapy group (HR: 0.34, 95% CI: 0.19–0.61, *P* < 0.001, eFig. 1C). In contrast, in patients with previous myocardial infarction, the risk of fatal or nonfatal myocardial infarction was nonsignificantly higher in the early revascularization, PCI, and CABG groups than in intensive the medical therapy group (HR: 1.31, 95% CI: 0.88–1.95, *P* = 0.17, eFig. 1A; HR: 1.35, 95% CI: 0.83–2.20, *P* = 0.23, eFig. 1B; HR: 1.25, 95% CI: 0.64–2.45, *P* = 0.51, eFig. 1C, respectively). Similar results were observed regarding the risk of cardiac or all-cause death, although there were no significant differences (eFigs 3–5).

**Figure 3:**
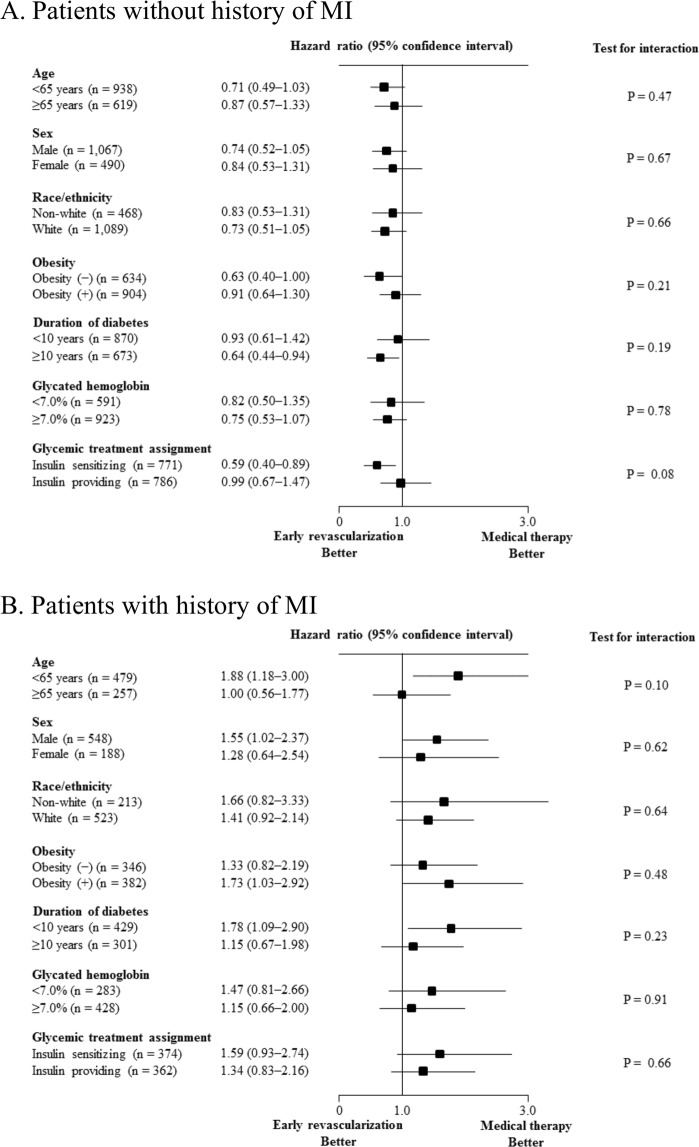
Association between the cardiac treatment strategy and the risk of cardiac events in patient subgroups. Associations in patients without (**A**) and with history of myocardial infarction (**B**). MI, myocardial infarction. Cox proportional hazard analyses were performed to calculate hazard ratios and P values for major cardiac events in the revascularization group were compared to the medical therapy group in the various subgroups.

The associations between the cardiac treatment strategy and major cardiac event risk in the subgroups are shown in Fig. 3. In patients without previous myocardial infarction, no significant interactions were found. Similarly, in patients with previous myocardial infarction, no significant interactions were observed. Contrary to patients without previous myocardial infarction, among those with previous myocardial infarction, the risk of major cardiac events within these subgroups seemed to be higher in the early revascularization group.

## Discussion

The present study demonstrated that patients without history of myocardial infarction had a lower risk of major cardiac events when they received revascularization, particularly CABG, than when they received only intensive medical therapy. In contrast, patients with history of myocardial infarction were at a higher risk of major cardiac events upon receiving revascularization than only intensive medical therapy. Significant interaction was found between the cardiac treatment strategy and the previous history of myocardial infarction, even in patients assigned to the CABG stratum, which were randomly assigned to the CABG or medical therapy groups. Similarly, although the risk of myocardial infarction in patients without previous myocardial infarction was lower in the CABG group than in the intensive medical therapy group, it was nonsignificantly higher in the revascularization group.

Several studies have previously compared revascularization strategies such as CABG and PCI to treat diabetic patients with CAD^19–22^. The Bypass Angioplasty Revascularization Investigation trial, performed in the era of PCI therapy using old balloon angioplasty, found that the 5 year survival of diabetic patients with CAD was significantly higher following CABG than PCI^19^. The Arterial Revascularization Therapy Study I and II investigators demonstrated that at 5 year follow-up, CABG had a superior efficacy preventing cardiovascular events in diabetic patients with CAD than PCI using bare-metal or drug-eluting stent^20^. In the Future Revascularization Evaluation in Patients with Diabetes Mellitus: Optimal Management of Multivessel Disease (FREEDOM) trial, which was performed at 140 international centers and included 1,900 diabetic patients with CAD, demonstrated that patients who underwent CABG had significantly lower rates of the composite primary outcome of myocardial infarction, stroke, or all-cause death than did those undergoing PCI with a drug-eluting stent^22^. The results of the FREEDOM trial were similar at all levels of angiographic complexity according to the SYNTAX score, which suggested that CABG was better than PCI for diabetic patients with CAD, regardless of the coronary disease complexity. Taken together, these results indicate that CABG may be the preferred therapy for improved outcomes in diabetic patients with CAD, as compared with PCI even using drug-eluting stent^23–25^. Meanwhile, few studies have previously compared revascularization and intensive medical therapy in diabetic patients with CAD. In the BARI 2D trial, the risk of mortality and major cardiovascular events among type 2 diabetic patients with CAD was similar for those undergoing early revascularization and those undergoing intensive medical therapy alone^7^. The trial also showed that the risk of death and a composite endpoint of death and major cardiovascular events was not significantly different between the PCI and intensive medical therapy, whereas the CABG was associated with a significant reduction of major cardiovascular events, as compared with intensive medical therapy. However, although a recent study reported that early revascularization in patients with a history of myocardial infarction or pathologic Q waves yielded no benefit^26^, the present study revealed that early revascularization may be even harmful for those with previous myocardial infarction, regardless of the cardiac procedures. The different results between the two studies may be attributed to the differences of the prespecified definitions of the myocardial infarction history and outcomes. In addition, the present study revealed that, even in the CABG stratum undergoing CABG as revascularization, significant interaction was found between the treatment strategy and the myocardial infarction history. Conversely, consistent with previous studies, CABG was a preferred therapy for diabetic patients with CAD and no history of myocardial infarction, compared with intensive medical therapy. The risk of revascularization in patients with history of myocardial infarction may match or surpass the benefits of reperfusion. The results of the present study indicate that the incidence of major cardiac events within 1 year of follow-up was particularly high in patients receiving early revascularization, including CABG, which suggests a risk of cardiac procedure-associated events. Diabetic patients often have multivessel coronary disease and coronary microvascular dysfunction^27^. Because diabetic patients with history of myocardial infarction already have suffered serious myocardial damage, the hemodynamic changes and coronary or myocardial damages associated with those occurring during revascularization may easily lead to subsequent adverse cardiac events. The exact reason remains unknown, and further investigations are warranted to confirm these findings.

This study has several limitations to report. First, the present study was a post-hoc and non-prespecified analysis of a randomized controlled trial. Therefore, these results are required to be confirmed by a future randomized controlled trial investigating major cardiac events as the primary outcome. Second, the BARI 2D trial included patients undergoing PCI with a bare-metal stent or no stent: 34.7% of the patients received a drug-eluting stent, and 56.0% received a bare-metal stent; the other 9.3% did not receive a stent^7^. The results may differ for patients who underwent PCI and those who underwent PCI with drug-eluting stent. Third, the reason for the observed association between cardiac treatment and myocardial infarction history remains to be explained by further clinical trials. Fourth, the number of patients undergoing each type of revascularization (PCI or CABG) was small and assigned at the physician’s discretion, which limited the analysis performed. Fifth, the present study could not evaluate CAD-associated symptoms, and further studies are required to evaluate the benefits of each cardiac treatment strategy to improve those symptoms in diabetic patients with CAD.

The present study demonstrates that in type 2 diabetic patients with CAD, those without previous myocardial infarction were at a lower risk of major cardiac events when treated with early revascularization, particularly CABG, than those receiving intensive medical therapy alone. Conversely, type 2 diabetic patients with CAD and previous history of myocardial infarction were at a higher risk of major cardiac events following early revascularization.

## Supplementary information

Supplemental Figures
