# Supplementary material for: Optimal cardiac strategy based on the history of myocardial infarction in type 2 diabetic patients with coronary artery disease
Source: Sci Rep. 2019 Mar 5;9:3502. doi: 10.1038/s41598-019-39857-0 (PMC6400970; doi:10.1038/s41598-019-39857-0)
Supplement: Supplementary file 1 — Supplemental Figures [file 41598_2019_39857_MOESM1_ESM.docx]

**Optimal cardiac strategy based on the history of myocardial infarction in type 2 diabetic patients with coronary artery disease**

Tetsuro Tsujimoto and Hiroshi Kajio

**Supplemental Figures**


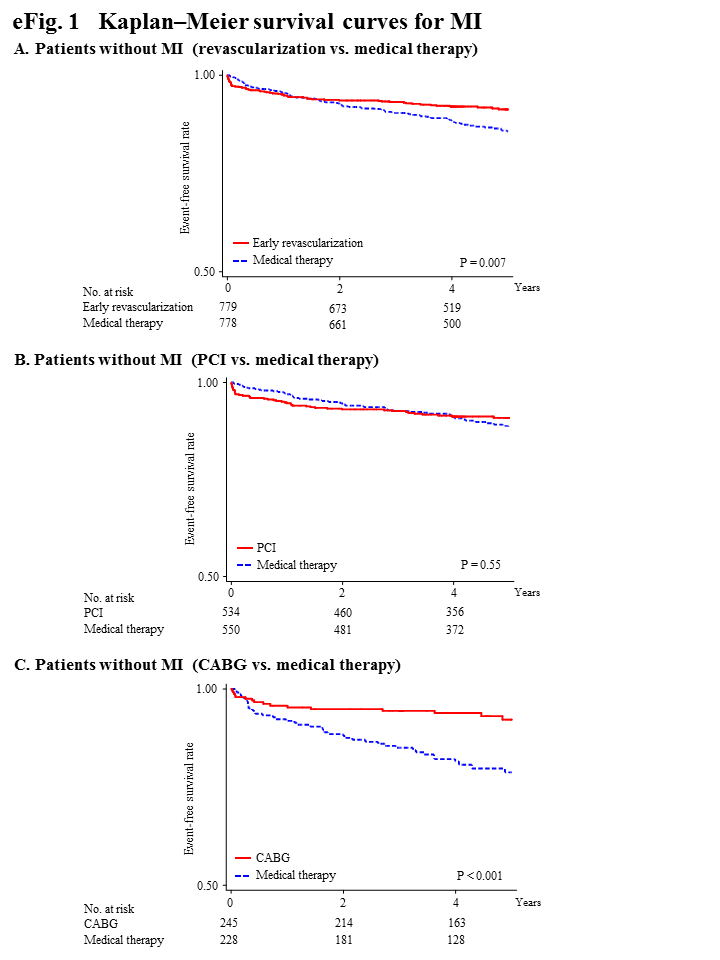


**eFigure 1. Kaplan–Meier survival curves for myocardial infarction in patients without history of myocardial infarction**

Rates of freedom from myocardial infarction: early revascularization vs. medical therapy (A), PCI vs. medical therapy (B), and CABG vs. medical therapy (C). Cox proportional hazard analyses were performed to calculate hazard ratios and P values for myocardial infarction in the revascularization (all, PCI, or CABG) group were compared to the medical therapy group in patients without history of myocardial infarction.

PCI, percutaneous coronary intervention; CABG, coronary artery bypass graft surgery.

**
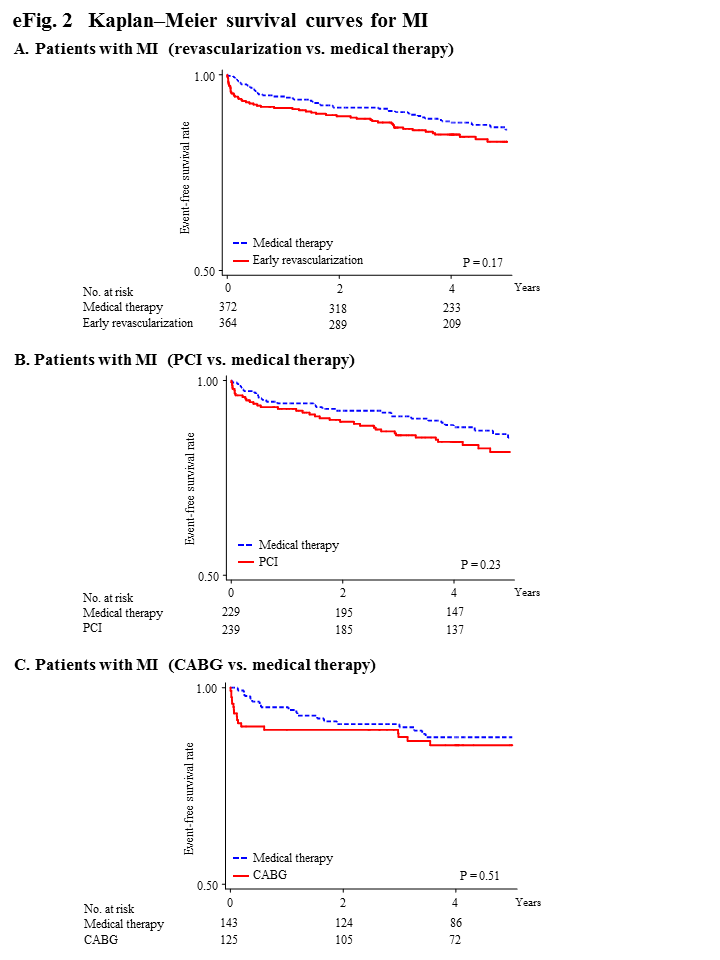
**

**eFigure 2. Kaplan–Meier survival curves for myocardial infarction in patients with history of myocardial infarction**

Rates of freedom from myocardial infarction: early revascularization vs. medical therapy (A), PCI vs. medical therapy (B), and CABG vs. medical therapy (C). Cox proportional hazard analyses were performed to calculate hazard ratios and P values for myocardial infarction in the revascularization (all, PCI, or CABG) group were compared to the medical therapy group in patients with history of myocardial infarction.

PCI, percutaneous coronary intervention; CABG, coronary artery bypass graft surgery.

**
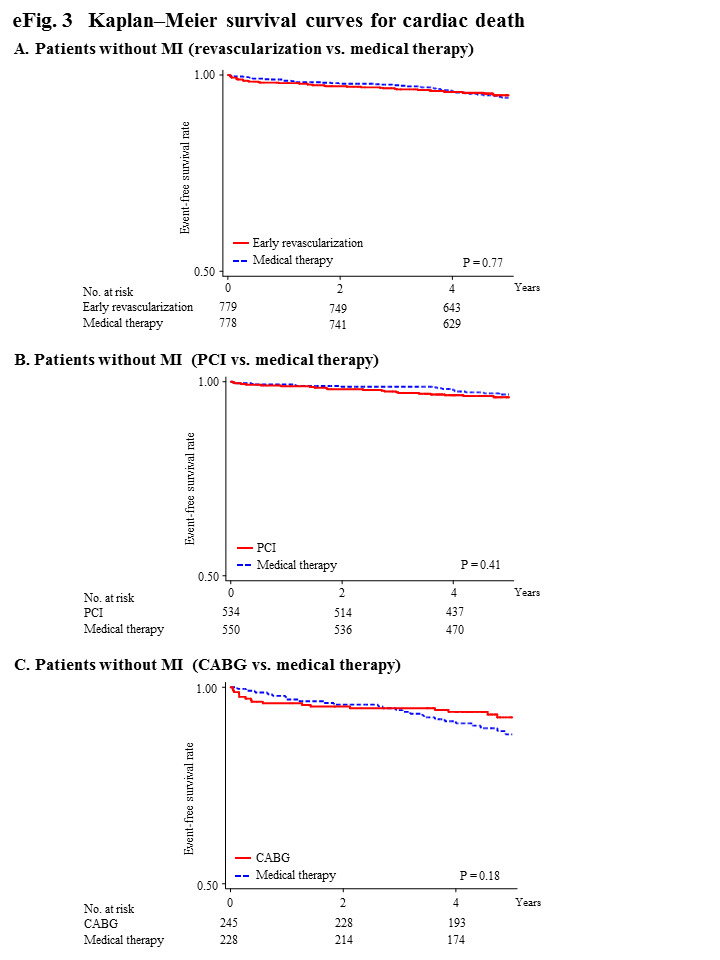
**

**eFigure 3. Kaplan–Meier survival curves for cardiac death in patients without history of myocardial infarction**

Rates of freedom from cardiac death: early revascularization vs. medical therapy (A), PCI vs. medical therapy (B), and CABG vs. medical therapy (C). Cox proportional hazard analyses were performed to calculate hazard ratios and P values for cardiac death in the revascularization (all, PCI, or CABG) group were compared to the medical therapy group in patients without history of myocardial infarction.

PCI, percutaneous coronary intervention; CABG, coronary artery bypass graft surgery.

**
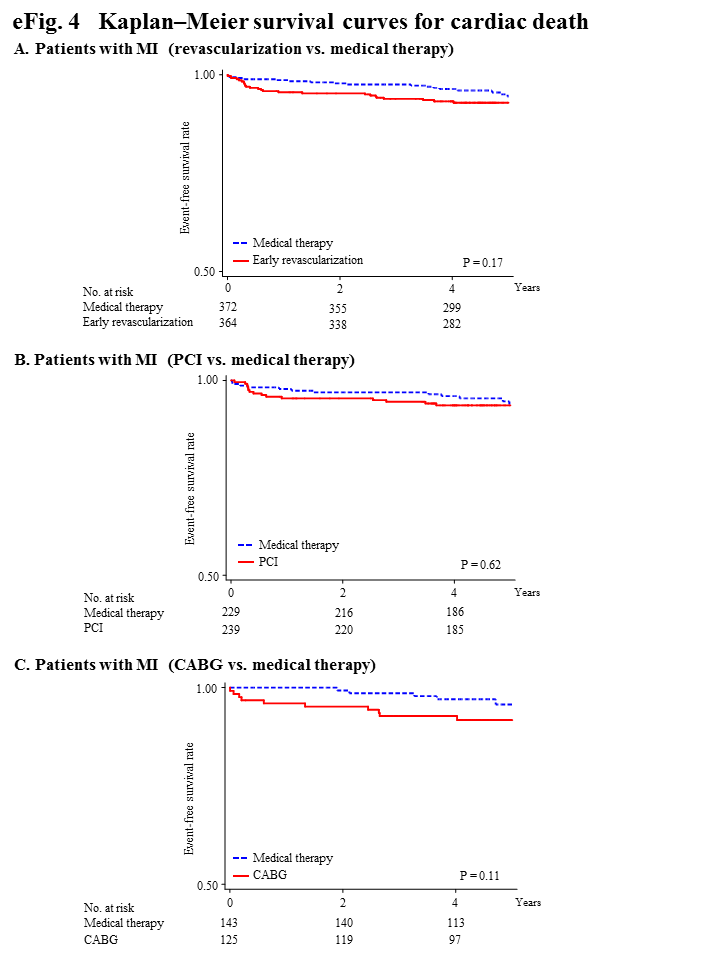
**

**eFigure 4. Kaplan–Meier survival curves for cardiac death in patients with history of myocardial infarction**

Rates of freedom from cardiac death: early revascularization vs. medical therapy (A), PCI vs. medical therapy (B), and CABG vs. medical therapy (C). Cox proportional hazard analyses were performed to calculate hazard ratios and P values for cardiac death in the revascularization (all, PCI, or CABG) group were compared to the medical therapy group in patients with history of myocardial infarction.

PCI, percutaneous coronary intervention; CABG, coronary artery bypass graft surgery.


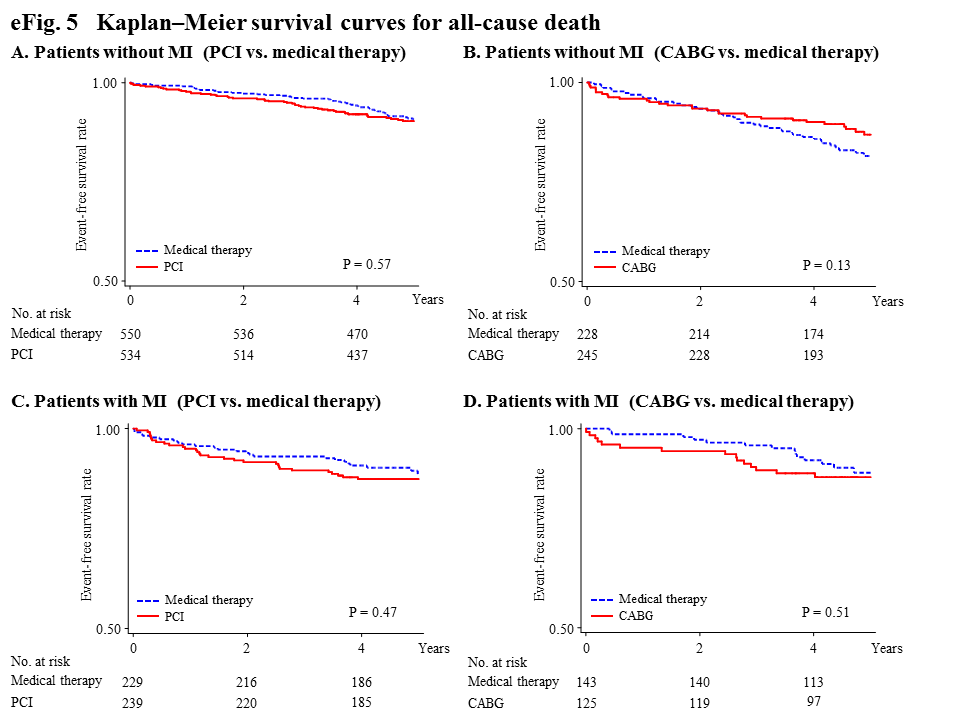


**eFigure 5. Kaplan–Meier survival curves for all-cause death in patients without and with history of myocardial infarction**

Rates of freedom from all-cause death: PCI vs. medical therapy (A) and CABG vs. medical therapy (B) in patients without history of myocardial infarction and PCI vs. medical therapy (C) and CABG vs. medical therapy (D) in patients with history of myocardial infarction. Cox proportional hazard analyses were performed to calculate hazard ratios and P values for all-cause death in the PCI or CABG group were compared to the medical therapy group in patients without and with history of myocardial infarction.

PCI, percutaneous coronary intervention; CABG, coronary artery bypass graft surgery.
